# Ultrasound—assisted periareolar oncoplastic approach in breast surgery: a focus on surgical technique

**DOI:** 10.3389/fsurg.2025.1623894

**Published:** 2025-09-12

**Authors:** Alessandro De Luca, Domenico Tripodi, Lucio Fortunato, Federica Pediconi, Claudio Cannistrà, Nicola Rocco, Massimo Vergine, Maria Ida Amabile

**Affiliations:** ^1^ Department of Surgery, Sapienza University of Rome, Rome, Italy; ^2^ Breast Center, Azienda Ospedaliera San Giovanni-Addolorata, Rome, Italy; ^3^ Department of Radiological, Oncological and Pathological Sciences, Sapienza University of Rome, Rome, Italy; ^4^ Plastic and Reconstructive Surgery Unit, Centre Hospitalier Universitaire Bichat Claude-Bernard, Paris, France; ^5^ Department of Advanced Biomedical Sciences, University Federico II, Naples, Italy

**Keywords:** breast cancer, oncoplastic breast surgery, breast conservative surgery, breast ultrasound, patient satisfaction, aesthetic outcome

## Abstract

**Introduction:**

Breast conservation surgery (BCS) combined with post-operative radiotherapy is the standard and preferred treatment for early-stage breast cancer (eBC), offering survival outcomes comparable to mastectomy while improving body image and quality of life. Oncoplastic breast surgery (OBS) has evolved from BCS to allow more extensive tissue removal while maintaining oncological safety and reducing the risk of post-surgical deformities. The ultrasound (US)-assisted periareolar approach in breast surgery offers several potential benefits, including reduced scarring, improved cosmetic outcomes, and enhanced surgical precision, particularly for non-palpable or small lesions, and potentially better nipple sensation preservation. This study aim to describe an US-assisted periareolar OBS approach for eBC patients with small to moderate breast ptosis.

**Methods:**

Here we present a focus on surgical technique consisting in OBS combining a US-assisted periareolar approach with volume displacement in small- to moderate- ptosic breasts. Margin resection adequacy, surgical complications and patient satisfaction using the Breast-Q questionnaire were assessed.

**Results:**

Thirty-two patients were considered. A negative margin of excision was achieved in all cases, and patients routinely received post-operative hypofractionated radiotherapy. Seroma was the most common complication (12.5%), while breast fat necrosis and minor wound infections occurred in 6% and 3% of cases, respectively. At a median follow-up of 12 months (range 6–18), post-treatment breast retraction occurred in 3 patients (9%), all of whom underwent fat grafting to improve outcomes. The average satisfaction score as determined by Breast-Q module was 78.6, rising to 81.3 for those who underwent contralateral mammaplasty.

**Discussion:**

The combination of imaging, the use of oncoplastic surgical techniques and an appropriate post-operative management may provide the surgeon new tools for the treatment of eBC. In selected cases, the US-assisted periareolar oncoplastic approach is a versatile technique that can be easily adapted for tumors in any location of the breast.

## Introduction

Breast conservation surgery (BCS) followed by post-operative radiotherapy represents the optimal locoregional treatment for the majority of patients with early-stage breast cancer (eBC), offering survival rates equivalent to those of mastectomy while preserving body image and significantly improving quality of life (1, 2).

It is well established that local recurrence rates after BCS combined with radiotherapy are comparable to those observed after mastectomy (2–4). However, BCS is associated with superior aesthetic and patient-reported outcomes, which can be further enhanced through the application of oncoplastic surgical techniques (5–7). Oncoplastic breast surgery (OBS) has been increasingly adopted in clinical practice as it enables wider resections while maintaining oncological safety (8). This approach helps avoiding unnecessary mastectomies and simultaneously reduces the risk of post-operative breast deformities and asymmetries (8, 9). The OBS allows for the removal of a larger volume of breast tissue (10, 11) and facilitates immediate reconstruction using plastic surgery principles. This can be achieved through either volume-displacement techniques, which involve the mobilization and reshaping of local dermo-glandular flaps (12), or volume-replacement strategies, where breast volume is restored using autologous tissue via various flap techniques (13, 14).

The increasing emphasis on aesthetic outcomes has led to growing interest in advanced oncoplastic methods (10–15), with many breast surgeons seeking additional training in reconstructive techniques or fostering closer collaboration with plastic surgery teams to improve the cosmetic results of BCS (16, 17).

In this context, the ultrasound (US)-assisted periareolar approach offers notable advantages. By integrating high-resolution intraoperative ultrasound with a minimally invasive periareolar access, this technique allows for precise tumor localization and excision with optimal margin control, all while preserving the natural breast contour and minimizing visible scarring. This approach not only supports oncological safety but also enhances cosmetic outcomes—particularly in cases involving small- to medium-sized tumors in the central or peri-areolar breast region. Furthermore, the use of ultrasound guidance reduces the likelihood of re-excision and improves intraoperative decision-making, ultimately contributing to greater patient satisfaction and overall quality of care.

In this study we present a focus on surgical technique consisting in OBS combining a US-assisted periareolar approach with volume displacement in small- to moderate- ptosic breasts, inspired by the Benelli technique described for aesthetic purposes (18).

## Materials and methods

A retrospective evaluation was conducted on consecutive patients who underwent OBS using an US-assisted periareolar approach at our Breast Center (Local Ethics Committee Protocol No. 0301/2021) in breast cancer patients with small- to medium- breast size and mild to moderate ptosis. We documented that patients with pathological skin involvement or tumors located more than 8 cm far from the nipple-areola complex were not treated with this approach. The study focused on patients with eBC with a follow-up period ranging from 6 to 18 months (median follow-up 11 months).

The surgical procedures utilized volume displacement techniques. In particular, the US-assisted periareolar approach was applied to facilitate tumor excision while reshaping the remaining breast tissue to preserve aesthetic outcomes.

Post-operative evaluations were carried out by two experienced oncoplastic breast surgeons, ensuring a comprehensive and standardized assessment of both clinical and cosmetic results. Patient satisfaction was assessed using the “Satisfaction with Outcome” module of the internationally validated BREAST-Q questionnaire (19), which is routinely administered during follow-up visits at our Center before beginning adjuvant radiotherapy. The questionnaire was completed three months post-operatively.

### Surgical technique description—the US-assisted periareolar approach

This oncoplastic surgical technique is structured around two essential pre-operative phases: a detailed breast ultrasound assessment and a precise pre-operative skin marking process.

The breast ultrasound is performed in both the supine and standing positions to ensure accurate identification of the area to be excised.

**Figure 1:**
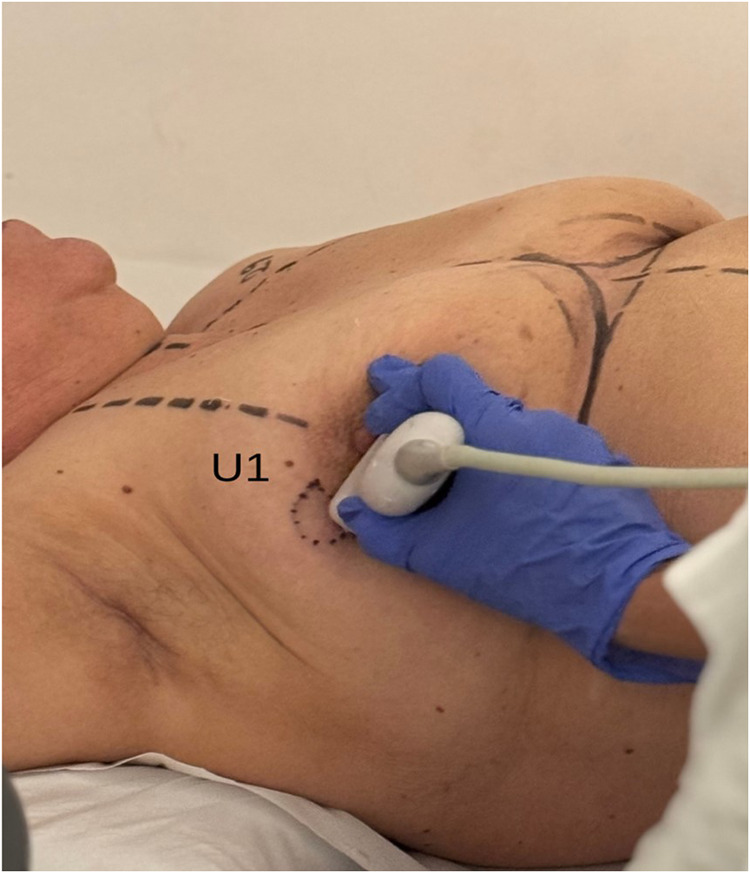
Breast ultrasound exam with patient in supine position (U1). Multifocal tumor was localized in the outer quadrants of the right breast.

**Figure 2:**
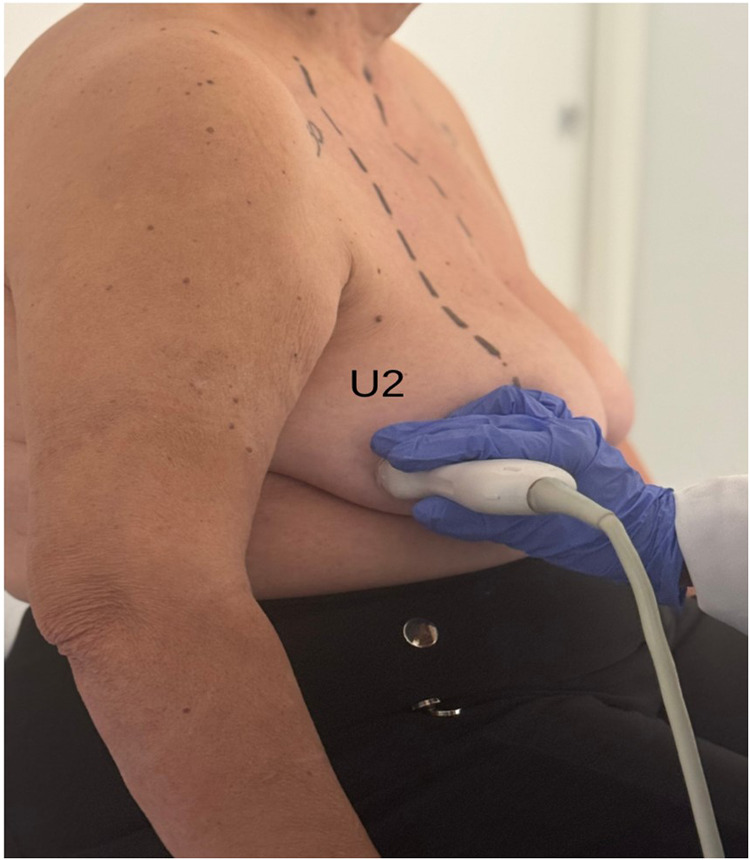
Breast ultrasound exam with patient in standing position (U1). Multifocal tumor was localized in the outer quadrants of the right breast.

• In the supine position, the tumor is located and marked, and its bidimensional measurements are recorded (**U1**, Figure 1).
• In the standing position, additional markings are made to account for tumor displacement due to breast ptosis, integrating this information into the surgical planning (**U2**, Figure 2).

**Figure 3:**
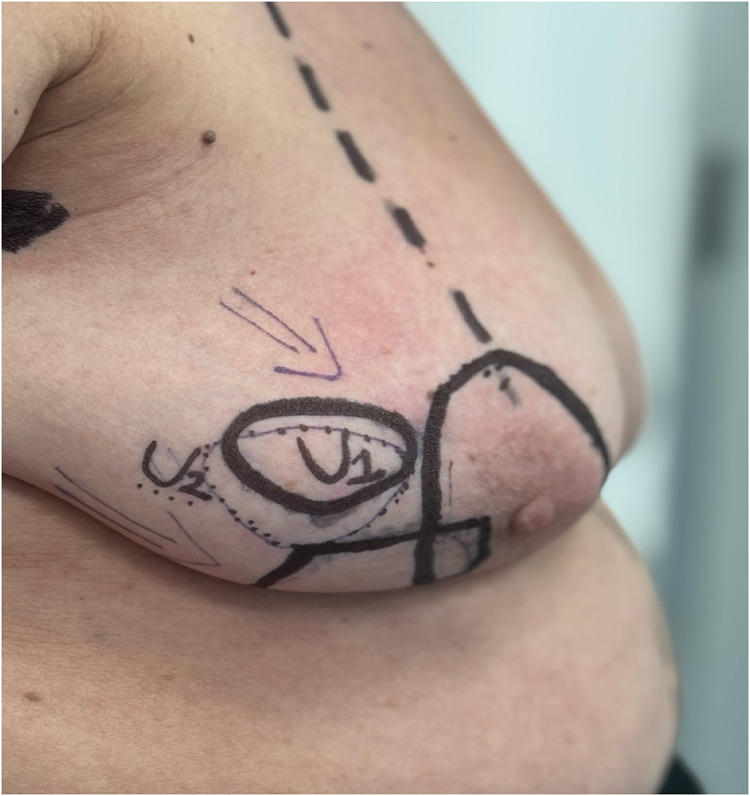
U1 and U2 breast markings. Multifocal tumor was localized in the outer quadrants of the right breast.

The definitive excision area is determined by combining the data from both positions (Figure 3).

Pre-operative skin markings are carried out in the patient's room while standing, facilitating anatomical accuracy and natural breast contour assessment.

**Figure 4:**
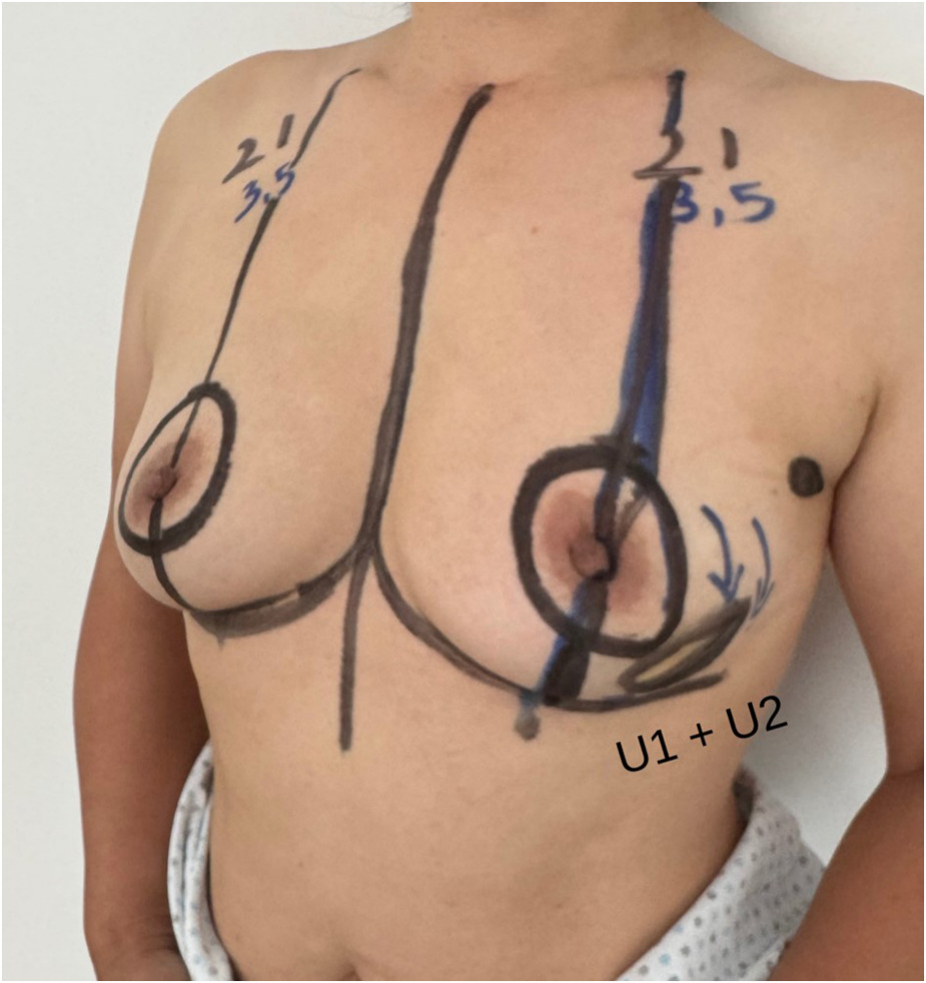
Preoperative drawing. Unifocal tumor was localized in the inferior external quadrant of the left breast.

Two concentric circular markings are applied:

• An inner circle outlining the periareolar border.
• An outer circle, whose placement is guided by ultrasound findings, tailored to the tumor location, breast volume, and degree of ptosis (Figure 4). This outer marking can be either concentric or eccentric to the nipple-areola complex and is limited to a maximum diameter of 6 cm.

**Figure 5:**
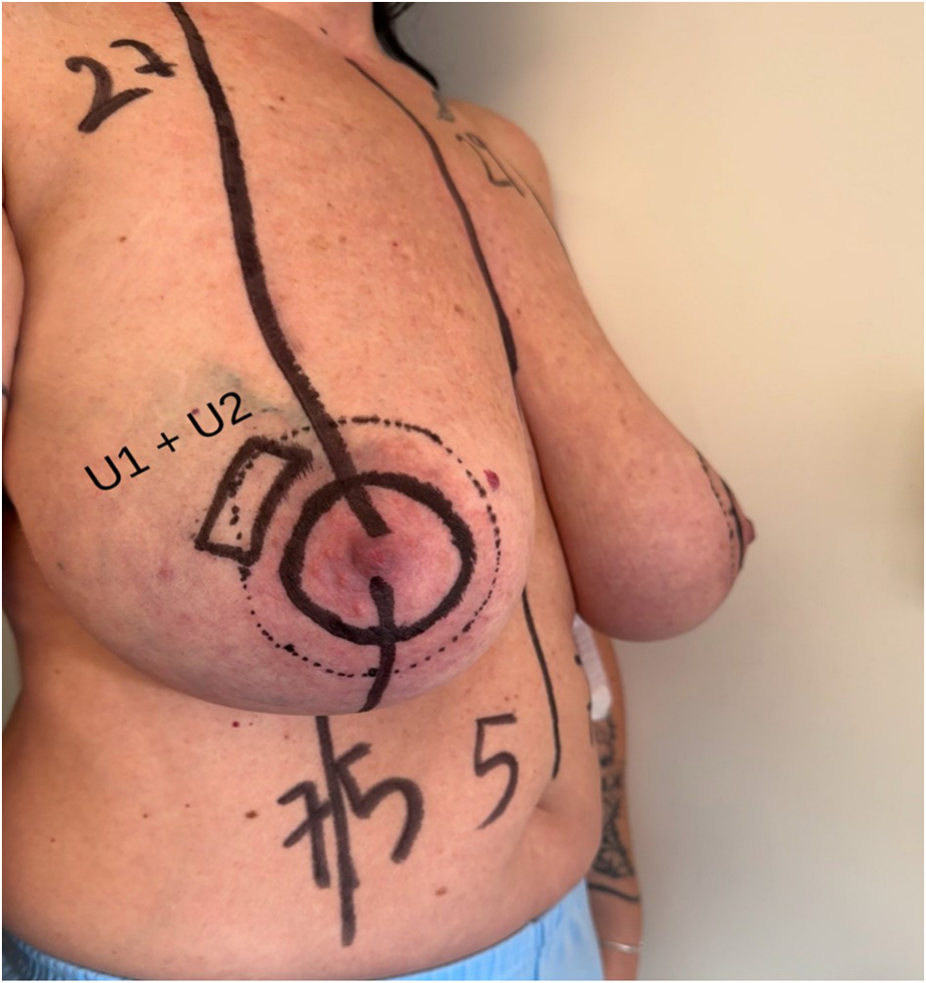
Preoperative drawing. Breast unifocal tumor was localized in the upper external quadrant of the right breast.

Reduction of the areola is performed only if its diameter exceeds 6 cm (Figure 5). Once the markings are completed, both breasts are prepped in the surgical field to allow intraoperative comparison for symmetry.

The patient is positioned on the operating table in a way that permits both supine and upright evaluations, optimizing reshaping and symmetry outcomes.

When the tumor is in close proximity to the areola, the overlying skin is removed *en bloc* with the lesion. If this is not required, we perform de-epithelialization of the skin between the two circular markings, sparing the dermal layer on the side opposite the tumor to preserve the vascular plexus of the nipple-areola complex.

Intraoperative margin analysis is conducted to ensure complete oncological resection. Following tumor removal, immediate glandular reconstruction and reshaping are undertaken.

**Figure 6:**
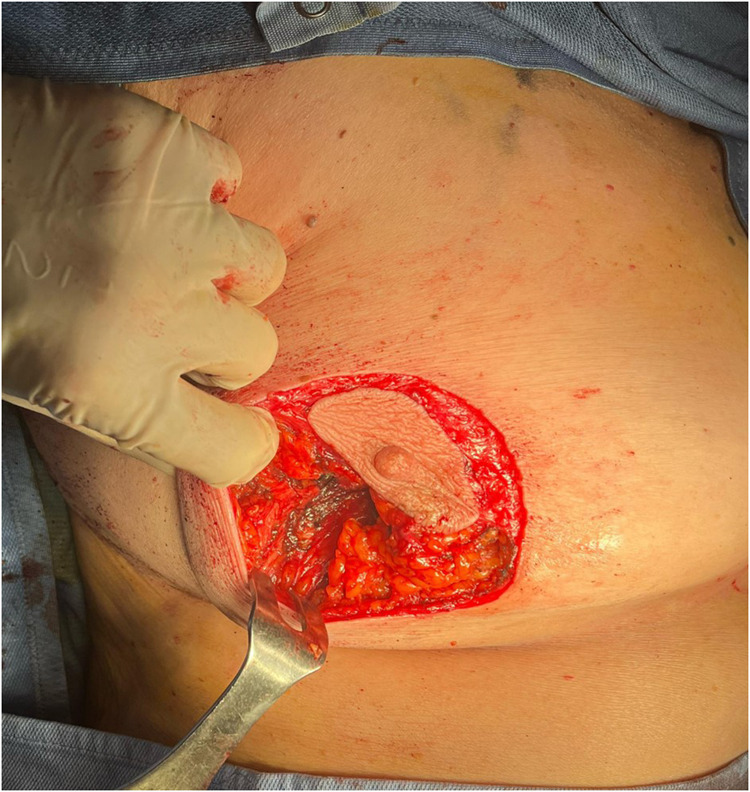
Subcutaneous undermining procedure. Multifocal tumor was localized in outer quadrants of the right breast.

**Figure 7:**
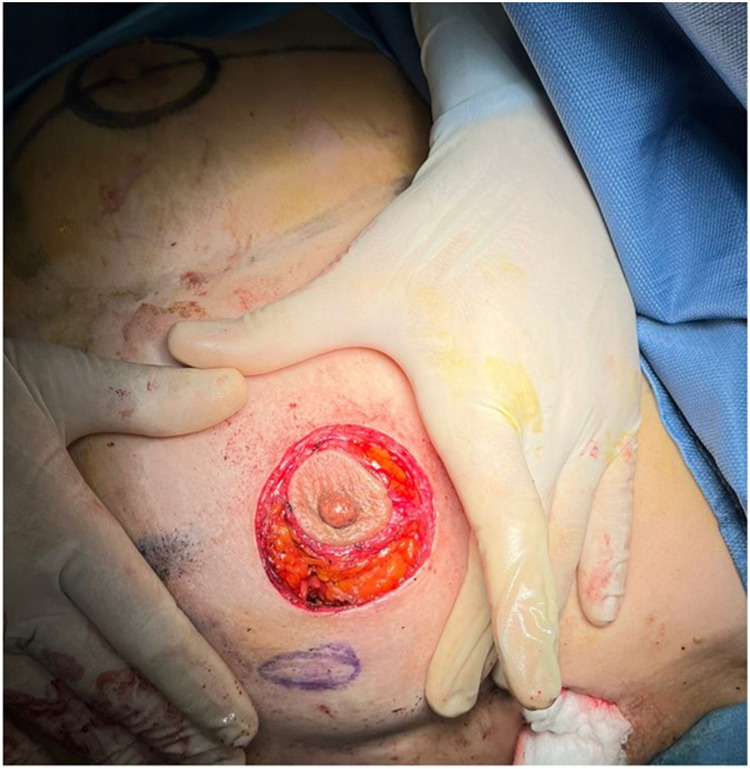
Subcutaneous undermining procedure. Unifocal tumor was localized in inferior external quadrant of the left breast.

A critical element of this technique is the extensive subcutaneous undermining performed prior to tumor excision. This approach, similar to that used in skin-sparing mastectomy, involves undermining 30%–50% of the breast envelope and may extend into either the upper or lower quadrants, depending on tumor location (Figures 6, 7).

The extent of subcutaneous dissection and glandular mobilization from the muscle-fascial plane is individualized for each patient, based on tumor location, resection volume, and breast size.

**Figure 8:**
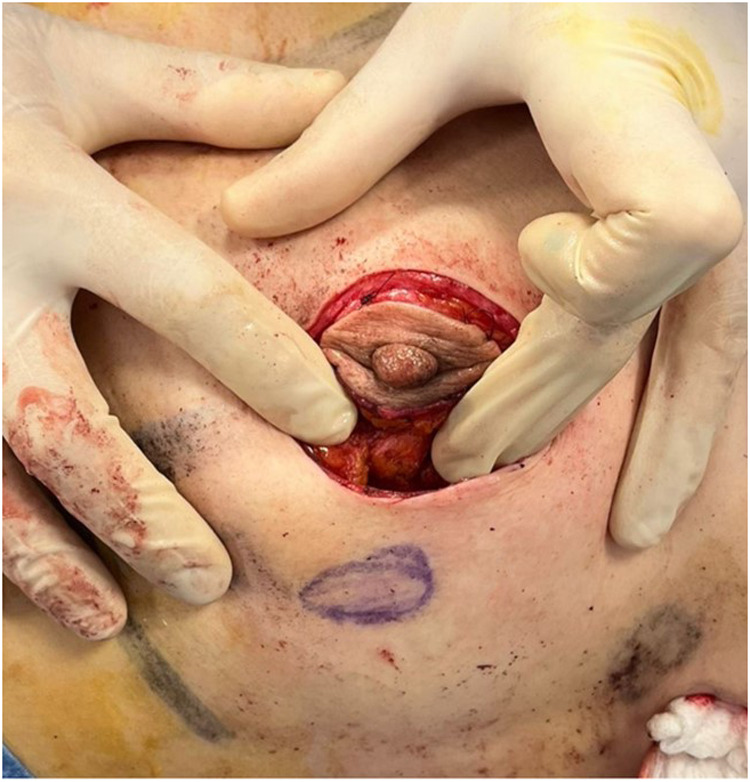
Round block technique. Unifocal tumor was localized in inferior external quadrant of the left breast.

**Figure 9:**
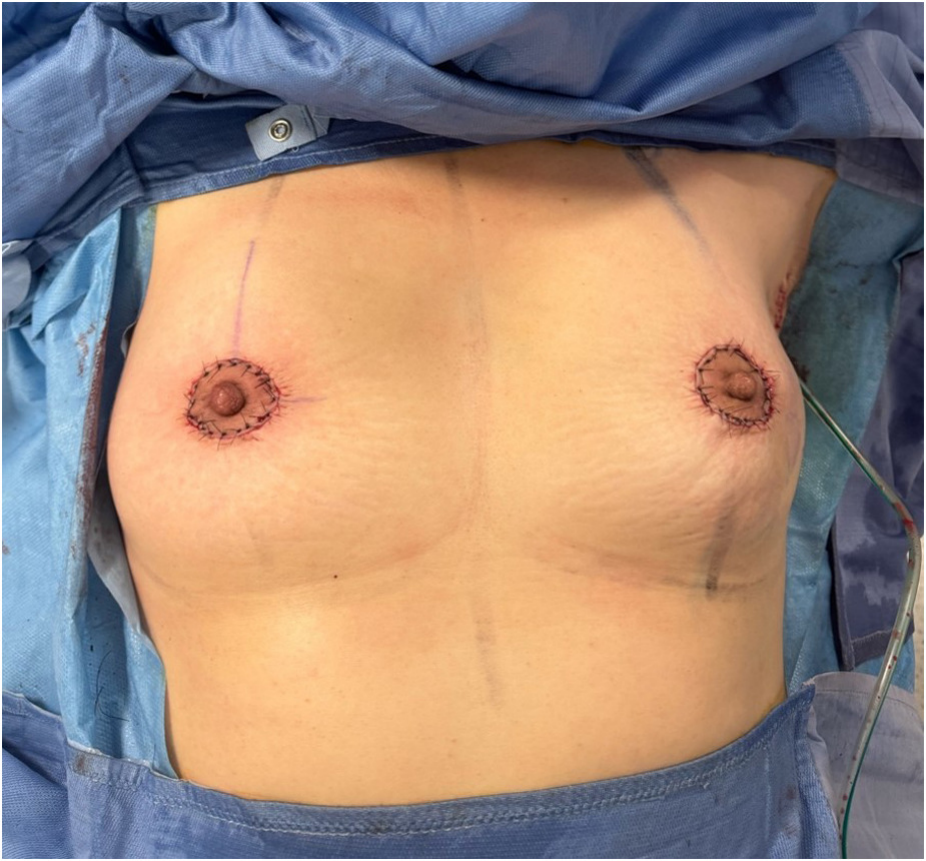
Post-operative result of ultrasound-assisted periareolar oncoplastic approach and contralateral mammoplasty in breast cancer patient with unifocal tumor in inferior external quadrant of the left breast.

The outer circular incision is reduced using a round block technique, approximating the deep dermal layer surrounding the areola with the retroareolar tissue and adjacent dermis. This technique yields a final scar confined to the periareolar region (Figures 8, 9), optimizing both cosmetic and oncological outcomes.

## Results

We initially considered a total of 40 potentially eligible eBC patients. Eight of these patients were excluded due to a radiological diagnosis of multicentric tumor or nipple-areola complex involvement. Therefore, 32 eBC patients undergone OBS with an US-assisted periareolar approach were considered, whose 18 patients also undergone a contralateral mammaplasty according to patient preference. The contralateral balancing surgery did not affect the surgical duration. It was performed with a 2-team approach, not lengthening the operating time.

The clinico-pathological characteristics of the patients are described in Table 1.

**Table 1:** Patients' clinico-pathological characteristics.

Clinico-pathological characteristics	Total (*N*)
Total	32
Age	
Mean	58.6 ± 9.8
Multifocal lesions (vs. Unifocal lesion)	
Yes	12.5% (*N* = 4)
No	87.5% (*N* = 28)
Clinical nodal stage before surgery	
N0	78% (*N* = 25)
N+	22% (*N* = 7)
Histological grade	
G1	12.5% (*N* = 4)
G2	47% (*N* = 15)
G3	40.5% (*N* = 13)
Histotype	
Ductal	81% (*N* = 26)
Lobular	12.5% (*N* = 4)
Other	6.5% (*N* = 2)
Biological subtype	
HER2-enriched	28% (*N* = 9)
Luminal A	25% (*N* = 8)
Luminal B	25% (*N* = 8)
Triple negative	22% (*N* = 7)
Type of breast surgery	
Monolateral	44% (*N* = 14)
Bilateral (controlateral mammaplasty)	56% (*N* = 18)
Type of lymph node treatment	
SLNB	78% (*N* = 25)
ALND	22% (*N* = 7)
Adjuvant radiotherapy	
Yes	100% (*N* = 32)
No	0

The mean age of patients was 58.6 ± 9.8 years and the most represented histology subtype was breast invasive ductal carcinoma (*N* = 26, 81%). No lesion required the removal of the nipple-areola complex.

The majority of the patients (*N* = 25, 78%) showed a cN0 status before surgery and all these patients (*N* = 25, 78%) underwent sentinel lymph node biopsy while the remaining underwent axillary lymph node dissection (*N* = 7; 22%).

The use of ultrasound guide shortened the surgical time because it allowed identifying the suspicious lesion/s faster and more effective than without the ultrasound guide.

In the post-operative phase, the seroma was the most prevalent complication observed (*N* = 4, 12.5%) (Table 2). We documented only 2 cases (6%) of breast fat necrosis and 1 case (3%) of minor wound infection, treated with specific antibiotic therapy (Table 2). No cases of major wound infection or NAC necrosis were reported. We obtained complete tumor excision (>2 mm free margins) in 100% of the patients.

**Table 2:** Post-operative complications.

Post-operative complications	Total (N)
Total	32
Seroma	
Yes	12.5% (*N* = 4)
No	87.5% (*N* = 28)
Breast Fat Necrosis	
Yes	6% (*N* = 2)
No	94% (*N* = 30)
Minor wound infection	
Yes	3% (*N* = 1)
No	97% (*N* = 31)
Major wound infection	
Yes	0
No	100% (*N* = 32)
NAC necrosis	
Yes	0
No	100% (*N* = 32)
Free margins	
Yes	100% (*N* = 32)
No	0
Post treatment breast retraction	
Yes	9% (*N* = 3)
No	93% (*N* = 29)
Fat Grafting	
Yes	9% (*N* = 3)
No	81% (*N* = 29)

**Figure 10:**
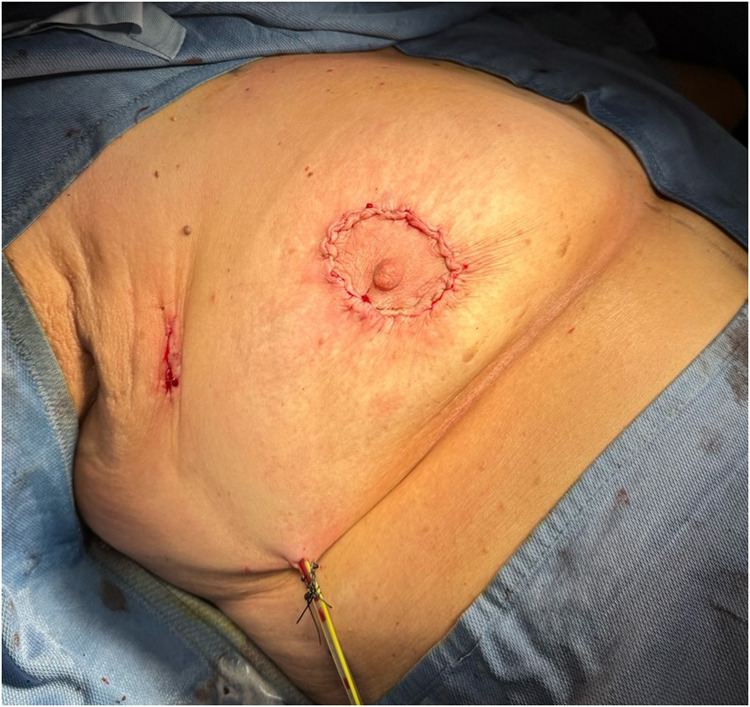
Post-operative result in ultrasound-assisted periareolar oncoplastic approach in breast cancer patient with multifocal tumor in outer quadrants of the right breast.

**Figure 11:**
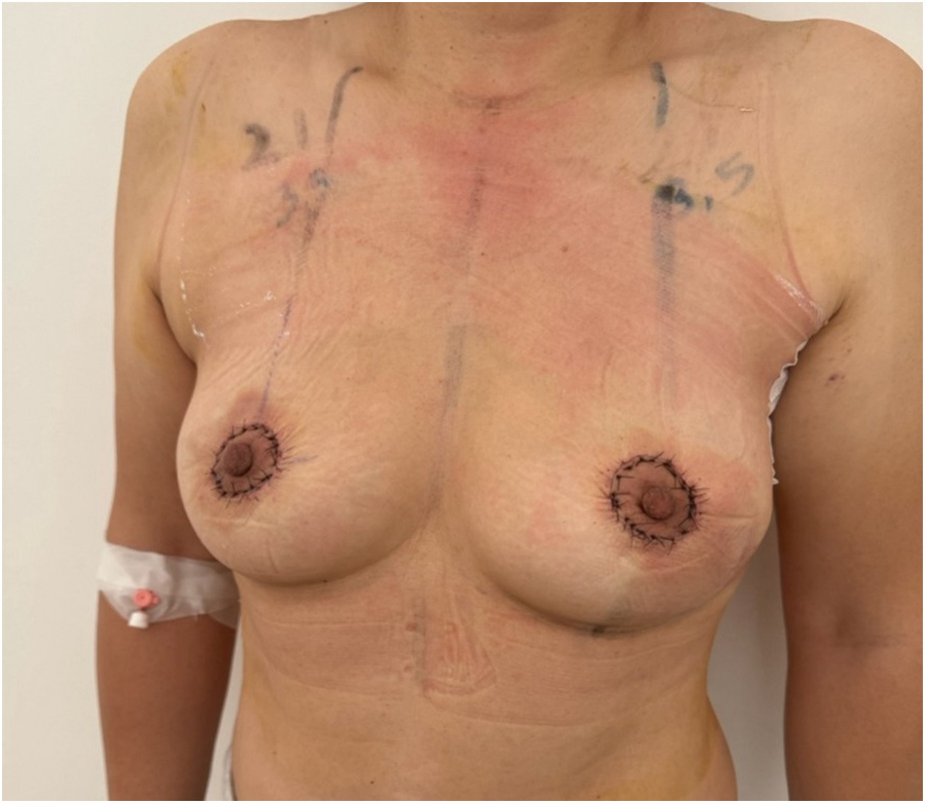
7-day post-operative time of ultrasound-assisted periareolar oncoplastic approach and contralateral mammoplasty in breast cancer patient with unifocal tumor in inferior external quadrant of the left breast.

All enrolled patients concluded adjuvant hypofractionated radiotherapy after surgery, which was conducted within 90 days from the surgery, as per our Center's internal guidelines. The study focused on patients with eBC, with a follow-up period ranging from 6 to 18 months (median follow-up: 11 months). The overall incidence of post-treatment breast retraction in our cohort was 9% (*N* = 3) and all these patients underwent further fat grafting procedure to improve the aesthetic and functional outcome (Table 2). We obtained a satisfaction score of 78.6 following the Breast-Q questionnaire administration, and a greater score of 81.3 when we considered only those patients who also underwent contralateral mammaplasty (Figures 10, 11). One patient complained of asymmetries and it was addressed further correcting with a fat grafting procedure.

## Discussion

Breast conservation therapy is widely recognized as a cornerstone in the treatment of eBC (1–4). Its integration with plastic surgery techniques represents a well-established approach that enables surgeons to perform wide excisions with negative margins while minimizing the risk of post-operative deformities (9, 16, 17).

In a systematic review, De La Cruz et al. reported low rates of positive surgical margins, re-excisions, and complications in patients with eBC treated with OBS, supporting its oncologic safety (8). Also our experience confirms that OBS allows for high rates of complete tumor excision—defined as clear margins >2 mm for ductal carcinoma *in situ* and no-ink on tumor for invasive carcinoma—without the need for reoperation. Furthermore, the use of US-assisted surgical techniques, combined with larger volumes of excised breast tissue, enabled the achievement of tumor-free margins in our patients, and this aspect may be as a rationale for implementing US-approach in clinical practice.

In this context, Giacalone et al. compared surgical margins obtained through conventional BCS vs. OBS, highlighting that margins of ≥10 mm are more frequently achieved with the oncoplastic approach (10). Similarly, Kaur et al. reported that OBS is associated with larger tissue resections and a reduced incidence of positive margins (11). Specifically, the US-assisted periareolar approach offers the dual benefit of extensive surgical access and precise intraoperative localization of the tumor, thereby increasing the likelihood of complete excision (10, 11, 20, 21).

Several studies have demonstrated that the integration of ultrasound guidance into BCS significantly improves the achievement of negative margins and reduces the need for reoperations (20–24), particularly in patients with palpable tumors. Positive margin rates as low as 3%–4% have been reported in such cases (22, 23).

Ultrasound-guided BCS is thus emerging as a reliable and effective technique for achieving negative surgical margins, with recognized feasibility and safety (20–24). Preoperative ultrasound allowed us to correct localize the lesions, supposing the feasibility of BCS with oncologically safe margins.

Patient satisfaction associated with the US-assisted periareolar approach was assessed using the BREAST-Q questionnaire (25, 26). Several studies have documented that OBS positively impacts quality of life in patients with eBC (27–31), considering that OBS is essential not only for achieving complete oncological resection but also for enhancing patient compliance with adjuvant therapies.

The main objective of OBS is to reshape the remaining breast tissue while preserving a natural and aesthetically pleasing breast contour. To this end, contralateral procedures are frequently performed to achieve optimal symmetry (25). Patient satisfaction and quality of life have become increasingly important indicators of surgical success, and these outcomes are closely linked to the extent of glandular and cutaneous undermining from the pectoralis muscle (10–12).

However, an aggressive undermining may lead to complications such as seroma formation and fat necrosis (32). Fat necrosis typically presents as a palpable mass with persistent firmness or as non-specific calcifications on mammography, usually measuring ≥1 cm (32, 33). We documented the incidence of seroma and fat necrosis was 12.5% and 6%, respectively. These complications occur principally in patients with predominantly fatty breasts on mammography (34).

In line with the literature (35–37), we found that the use of at least one surgical drain, appropriate antibiotic prophylaxis, and post-operative elastic compression bandaging contributed to a reduction in the incidence of seroma, fat necrosis, and surgical site infections.

Seroma rate may be significantly influenced by a myriad of surgical aspects, including but not limited to: a. extent of surgical undermining and dissection of the breast parenchyma; b. surgical technique, i.e., use of electrocautery vs. scalpel; c. whether drains are placed and for how long; d. the type of axillary procedures performed (i.e., axillary clearance or sampling vs. sentinel lymph node biopsy), as lymphorrhoea might be commonly mixed in or confused with serous collections, especially from axillary drains; e. patient-related factors (age, body mass index, certain comorbidities, smoking, etc.); f. use of compressing dressings or garments. In addition, judging by the fact that 30%–50% of the breast surface is undermined in the surgical approach, a considerable dead space is created. This may, in part, explain the seroma rate that we observed in our cohort.

The final aesthetic result of OBS depends on several factors, including age, comorbidity, tumor size and site, breast volume, and adjuvant treatments including radiotherapy (38, 39). Adjuvant radiotherapy is essential to reduce the risk of local recurrence (40) as well as determining late deformities through tissue fibrosis (41, 42). In our cohort 3 patients (9%) reported post-treatment breast retraction which required at least 2 fat-grafting sessions to improve the functional and aesthetic outcome (43–45). In order to minimize complications and optimize aesthetic outcomes, each clinical case should be managed by a multi-disciplinary team for a correct clinical and radiological assessment (46–48). Periareolar techniques are quite powerful as they can address small asymmetries of the nipple-areola complex. However, the main limitation is that the periareolar suture compresses the breast reducing its projection considerably, if the procedure is not accompanied by the placement of a breast implant which can help provide additional projection. Of course, placing an implant on an irradiated breast is commonly frowned upon by many surgeons due to the very high rate of complications which is upwards of 50% (49).

Furthermore, considering the patient-centered benefits, including reduced discomfort and fewer preoperative procedures, it suggests that US-assisted periareolar approach may be, in selected cases, a preferred method in managing early-stage breast lesions, particularly in high-volume cancer centers where resource optimization is critical.

We are aware that our description has limits: surely, the small cohort of considered patients and the brief follow-up period, which limits the generalizability of the findings, as well as the fact that long-term recurrence data are not yet available. We did not preoperatively collect BREAST-Q results. Assessing the BREAST-Q preoperatively is essential to establish a baseline for evaluating the true impact of surgery on patient-reported outcomes. The lack of pre-operative data makes it difficult to determine whether post-operative changes reflect improvement, decline, or no change at all. It also helps capture patient expectations, supports individualized counseling, and enhances the validity of clinical research through within-subject comparisons. By measuring both pre- and post-operative outcomes, clinicians can better assess the effectiveness of surgical interventions and continuously improve the quality of care. In addition, BREAST-Q was collected post-operatively at 3 months, before beginning adjuvant radiotherapy, and this may determine a bias when assessing aesthetic outcomes, as radiotherapy negatively affects aesthetic outcomes.

The median follow-up period was short for evaluating oncological outcomes. While early cosmetic results are informative, long-term recurrence data are not yet available for our patients. We did not perform power calculations and define the minimum number of cases performed without ultrasound to draw a comparison group and design a case-control study, matching patients in each group by demographic and oncological characteristics, and thus to find associations with tangible benefits of one technique compared to the other.

Here we present a focus on surgical technique consisting in OBS combining a US-assisted periareolar approach with volume displacement in small- to moderate- ptosic breasts. Further analysis needs to be presented with detailed descriptive data and to determine whether any number of variables may affect the clinical and/or aesthetic outcomes, which, in turn, would considerably strengthen the findings as a retrospective cohort study.

## Conclusions

The combination of imaging, the use of oncoplastic surgical techniques and an appropriate post-operative management may provide the surgeon new tools for the treatment of eBC. In selected cases, the US-assisted periareolar oncoplastic approach is a versatile technique that can be easily adapted for tumors in any location of the breast.

## Data Availability

The original contributions presented in the study are included in the article/Supplementary Material, further inquiries can be directed to the corresponding author.
